# Peptidylarginine Deiminase 2 Gene Polymorphisms in Subjects with Periodontitis Predispose to Rheumatoid Arthritis

**DOI:** 10.3390/ijms23179536

**Published:** 2022-08-23

**Authors:** Laura Massarenti, Christian Enevold, Dres Damgaard, Peter Riis Hansen, Morten Frisch, Niels Ødum, Søren Jacobsen, Claus Henrik Nielsen

**Affiliations:** 1 Institute for Inflammation Research, Center for Rheumatology and Spine Disease, Section 7521, Copenhagen University Hospital Rigshospitalet, 2200 Copenhagen, Denmark; 2 Department of Cardiology, Herlev-Gentofte Hospital, 2900 Hellerup, Denmark; 3 Department of Clinical Medicine, Faculty of Health and Medical Sciences, University of Copenhagen, 2200 Copenhagen, Denmark; 4 Department of Epidemiology Research, Statens Serum Institut, 2300 Copenhagen, Denmark; 5 LEO Foundation Skin Immunology Research Center, Department of Immunology and Microbiology, University of Copenhagen, 2200 Copenhagen, Denmark; 6 Copenhagen Lupus and Vasculitis Clinic, Center for Rheumatology and Spine Diseases, Section 4242, Copenhagen University Hospital Rigshospitalet, 2100 Copenhagen, Denmark; 7 Section for Periodontology, Department of Odontology, Faculty of Health and Medical Sciences, University of Copenhagen, 2200 Copenhagen, Denmark

**Keywords:** rheumatoid arthritis, peptidylarginine deiminases, anti-citrullinated protein antibodies (ACPAs), single nucleotide polymorphism, periodontitis

## Abstract

Epidemiologic studies have shown associations between periodontitis and rheumatoid arthritis (RA), but a causal relationship has not been established. Citrullination of gingival proteins by human peptidylarginine deiminases (PADs) or PAD from *Porphyromonas gingivalis* has been proposed to generate autoantigens in anti-CCP-positive RA. This study investigated whether the association between periodontitis and RA is influenced by single nucleotide polymorphisms (SNPs) in the genes encoding PAD2 and PAD4 that catalyze aberrant citrullination in RA and often are overexpressed in inflamed gingival connective tissue in subjects with periodontitis. The study included 137 RA patients and 161 controls with self-reported periodontitis. Periodontitis onset preceded RA onset by 13 years on average and was not associated with any of the SNPs investigated. In subjects with periodontitis, carriage of the minor alleles of rs2057094 and rs2235912 in *PADI2* significantly increased the risk of RA (odds ratios 1.42 [*p* = 0.03] and 1.48 [*p* = 0.02], respectively), and this effect was driven by the anti-CCP-negative RA patients. The minor alleles of these SNPs only increased risk of anti-CCP-positive RA in individuals with periodontitis and a history of smoking. These data suggest that individuals with periodontitis carrying the minor alleles of SNPs rs2057094, rs2076616 and rs2235912 in *PADI2* may be at increased risk of RA.

## 1. Introduction

Periodontitis is the most prevalent chronic inflammatory disease, affecting up to 50% of the adult population, and its frequency increases with age [1]. Several studies have shown an association between periodontitis and rheumatoid arthritis (RA) [2,3,4], but a causal link has not been shown [5,6]. Both diseases result in bone destruction [7,8,9,10] and share genetic and environmental risk factors, including smoking and certain HLA-DR allotypes containing an amino acid sequence known as the shared epitope in the peptide-binding groove [11,12,13,14].

Citrullination, the post-translational conversion of peptidylarginine into peptidylcitrulline catalyzed by peptidylarginine deiminases (PADs), plays a role in RA pathogenesis in the around 2/3 of RA patients who produce anti-citrullinated peptide antibodies (ACPAs) that can be detected in assays using cyclic citrullinated peptide (CCP) as antigen [15]. Citrullinated proteins can be found in gingival connective tissue in subjects with periodontitis [16,17,18], as well as in the synovium of RA patients [19,20,21]. Two periodontitis-associated bacteria, *Porphyromonas gingivalis* and *Aggregatibacter actinomycetemcomitans,* have been suggested to mediate citrullination of autoantigens that may contribute to the pathogenic processes in RA. *P. gingivalis* expresses a PAD (PPAD) and *A. actinomycetemcomitans* produces pore-forming leukotoxins (LtxA, LtxB, LtxC and LtxD) that facilitates the action of intracellular PADs of human leukocytes [22,23]. Both bacteria have also been demonstrated to promote the release of neutrophil extracellular traps (NETs) [23,24] and thus release of human PADs to the extracellular environment [25]. Indeed, the inflamed periodontium contains increased protein and mRNA levels of the two human PAD isoforms PAD2 and PAD4 that are thought to drive autoantigen citrullination in RA [18].

Several single nucleotide polymorphisms (SNPs) in the genes encoding PADs, including *PADI2* (e.g., rs2057094, rs2076616, rs2235912 and rs1005753) and *PADI4* (e.g., rs74058715, rs11203367, rs1748033 and rs2240335), have been shown to associate with RA in groups of different ethnicities [26,27,28,29]. To our knowledge, no *PADI* SNPs have been associated with periodontitis. We have recently demonstrated that SNPs in *PADI4* contribute to the cumulative risk of anti-CCP-positive RA, particularly in individuals carrying *HLA-DR4* [30]. In the present study, the association of *PADI4* and *PADI2* SNPs with RA in individuals with self-reported periodontitis was investigated.

## 2. Results

### 2.1. Demographic and Clinical Characteristics

Of the 434 patients with RA included in this study, 137 (32%) had self-reported periodontitis, and of the 524 healthy controls without RA, 161 (31%) had periodontitis. The patient and control groups were comparable with respect to age, sex, ethnicity, and smoking. Self-reported age at periodontitis onset was known for most RA cases (127/137 [93%]) and controls (146/161 [91%]).

The prevalence of self-reported periodontitis did not differ between RA patients and healthy controls, nor between anti-CCP-positive or anti-CCP-negative RA patients and healthy controls (Table 1).

However, in participants with both diagnoses, the onset of periodontitis preceded the onset of RA by an average of 13 (range −2; 52) years (Table 1). In 118 (86%) of those cases, periodontitis was diagnosed before RA.

### 2.2. Associations between PADI SNPs and Periodontitis

Having previously established that *PADI4* SNPs associate with RA in the same cohort [30], we examined whether SNPs in either *PADI2* or *PADI4* associated with periodontitis in the non-RA control group. None of the examined SNPs in *PADI2* (rs2057094, rs2076616, rs2235912 and rs1005753) or *PADI4* (rs74058715, rs11203367, rs1748033 and rs2240335) were significantly associated with periodontitis per se (data not shown).

### 2.3. Associations between PADI SNPs and RA in Subjects with Self-Reported Periodontitis

As periodontitis and *PADI* SNPs have been suggested to increase the risk of RA, in general, and of ACPA-positive RA, in particular, we examined the associations between *PADI* SNPs and RA in the subgroup of patients with self-reported periodontitis.

In patients with RA and controls with self-reported periodontitis, we found significant associations between two SNPs in *PADI2* (rs2057094 and rs2235912) and RA after adjustment for age, sex, and smoking status (Odds Ratio (OR) 1.42 [*p* = 0.03] and 1.48 [*p* = 0.02], respectively) (Table 2); rs2076616 showed a similar trend (OR 1.35, *p* = 0.08).

Analyses performed after stratification by anti-CCP status revealed that the observed association with RA were driven by the anti-CCP-negative patients. Thus, rs2057094, rs2076616 and rs2235912 associated with anti-CCP-negative RA (OR 1.75 [*p* = 0.02], 1.67 [*p* = 0.04], and 2.04, [*p* = 0.004], respectively), while no significant association with anti-CCP-positive RA was observed (Table 2).

Associations between RA and SNPs in *PADI4* were not statistically significant in the group with self-reported periodontitis (data not shown).

### 2.4. Associations between PADI SNPs and RA by Smoking Status

As smoking is also regarded as an important risk factor for ACPA-positive RA in particular [12,31], and *PADI* SNPs have been suggested to interact with smoking in the pathogenesis of RA [30,32], we examined the associations between SNPs in *PADI2* in relation to anti-CCP status and smoking status in the subgroup of patients with self-reported periodontitis.

**Figure 1:**
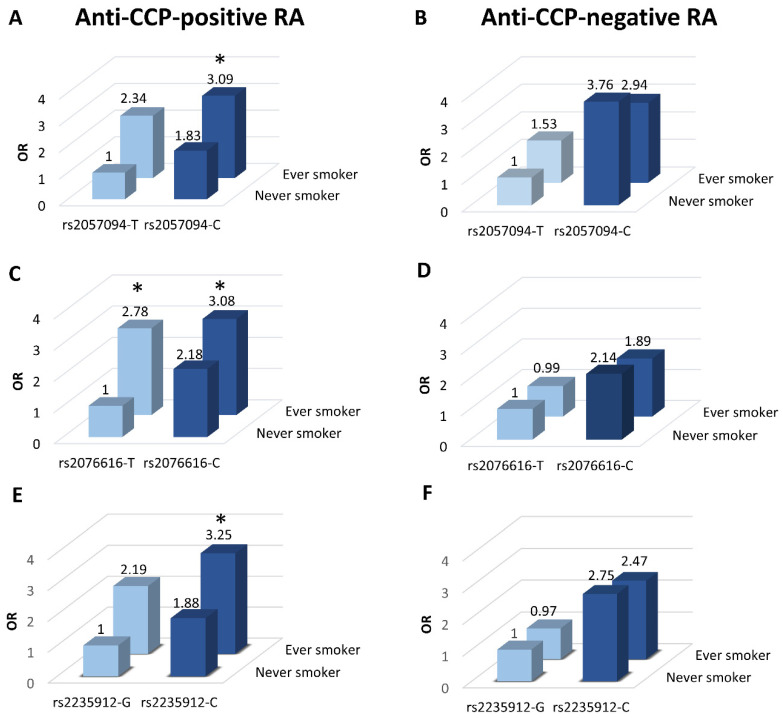
Influence of peptidylarginine deiminase gene 2 (*PADI2*) single nucleotide polymorphisms (SNPs) on the risk of rheumatoid arthritis (RA) among subjects with self-reported periodontitis. The influence of *PADI2* SNPs rs2057094 (**A**,**B**), rs2076616 (**C**,**D**) and rs2235912 (**E**,**F**) on risk of anti-CCP–positive (**A**,**C**,**E**) and anti-CCP–negative RA (**B**,**D**,**F**) is shown. Bars represent cumulative odds ratio (OR) due to homozygosity for the major allele (light blue bars) or carriage of the minor allele (dark blue bars) and smoking status. Bars marked with “1” denote reference conditions. * *p* < 0.05. All OR were adjusted for age and sex.

As shown in Figure 1A, the risk of anti-CCP-positive RA was increased in ever-smokers carrying the minor (C) allele of rs2057094 (OR 3.09, *p* = 0.03). On the other hand, the increased risk of anti-CCP-negative RA in subjects who carried the minor allele did not depend on smoking status (Figure 1B).

The risk of anti-CCP-positive RA was also increased in ever-smokers carrying the minor (C) allele of rs2076616 (OR 3.08, *p* = 0.02; Figure 1C), as well as in ever-smokers, who did not carry the minor (C) allele of this SNP (OR 2.78, *p* = 0.03; Figure 1C), while the risk of anti-CCP-negative RA did not depend on smoking status (Figure 1D).

Likewise, the risk of anti-CCP-positive RA was increased in ever-smokers carrying the minor (C) allele of rs2235912 (OR 3.25, *p*= 0.02; Figure 1E), while the risk of anti-CCP-negative RA was not affected by smoking status (Figure 1F).

Further adjustments of these analyses for carriage of *HLA-DR4* or *HLA-DR1* did not significantly change the results (data not shown).

## 3. Discussion

A link between periodontitis and RA has been demonstrated in several epidemiological studies [3,4], but a causal relationship has not been firmly established. In agreement with other studies [33,34], we found no difference in prevalence of periodontitis between anti-CCP-positive and anti-CCP-negative patients with RA. Interestingly, in the study participants with both RA and self-reported periodontitis, the onset of periodontitis preceded that of RA by an average of 13 years, suggesting that periodontitis may predispose to RA rather than vice versa.

Citrullination is considered a central process in the pathogenesis of anti-CCP-positive RA, and may also play a role in anti-CCP-negative RA. Thus, studies on SNPs in the genes encoding PAD2 and PAD4 have shown associations with both forms of RA [26,27,28,29,30]. The role of *PADI* polymorphism in periodontitis is not clear, but none of the *PADI2* or *PADI4* SNPs examined in this study associated with periodontitis.

In this study, we examined the association between *PADI* SNPs and RA in individuals with self-reported periodontitis. We observed that the minor alleles of *PADI2* SNPs rs2057094 and rs2235912 conferred increased overall risk of RA. Further stratification by anti-CCP status revealed that these SNPs did not associate significantly with anti-CCP-positive RA, but with anti-CCP-negative RA. On the other hand, we did not observe any association between *PADI4* SNPs and RA, irrespective of anti-CCP status. These findings suggest that citrullination by human PAD2 may play a role in the pathogenesis of anti-CCP-negative RA, particularly in subjects with a chronic inflammatory disease such as periodontitis. This is contrast with the general hypothesis that citrullination mainly matters in the pathogenesis of anti-CCP-positive RA.

As expected, we found that the risk of anti-CCP-positive RA was higher in smokers than in non-smokers, and we observed that carriers of the minor alleles of *PADI2* SNPs rs2057094, rs2076616 and rs2235912 had increased cumulative risk of anti-CCP-positive RA compared to non-carriers. As also expected from the well-established lack of association between smoking and anti-CCP-negative RA [31,35], smoking did not appear to synergize with *PADI2* SNPs in conferring risk of this RA subtype.

Little is known about the impact of *PADI2* polymorphisms on the function of PAD2. Of particular relevance for anti-CCP-positive RA, it can be speculated that the SNPs studied here encode PAD2 variants that more efficiently generate citrullinated autoantigens in the inflamed periodontal tissue where PAD2 is reportedly overexpressed [17,18]. An increased activation and release of PAD2 from neutrophils in periodontal tissue could be due to the leukotoxic activity of *A. actinomycetemcomitans*, which is often harbored by patients with periodontitis [36]. Of relevance for both anti-CCP-positive and anti-CCP-negative RA, increased NETs formation has been demonstrated in RA, independently of the presence of ACPAs [37], and a role for PAD2 in NETs formation has recently been demonstrated [38]. Thus, SNPs in *PADI2* may cause increased NETosis and consequent inflammatory effects. Indeed, the minor (C) allele of rs2235912, located in an intronic region, has been associated with decreased content of PAD2 in neutrophils, which may reflect increased PAD2 secretion and local inflammation [29]. Notably, rs2057094 and rs2076616, both intronic SNPs, have not, to our knowledge, been associated with alterations of the biological activity of PAD2.

A limitation of our study is the lack of detailed examination of the participants by a dentist including recording of periodontitis severity, but a recent meta-analysis showed that self-reported periodontal disease has acceptable validity in epidemiological studies [39]. Besides, the exact number of years by which the diagnosis of periodontitis preceded the diagnosis of RA should be interpreted with caution, because this was likely subject to recall bias. However, this does not change the fact that periodontitis appeared years before RA in individuals with both diseases. Moreover, our study cohort contained a relatively small number of subjects with periodontitis, and due to the exploratory nature of the study, we did not correct for multiple testing. Nevertheless, the fact that three of four examined *PADI2* SNPs in low to moderate linkage disequilibrium (LD) (r^2^ 0.22–0.66) showed significant associations with RA, in general, and with anti-CCP-negative RA, in particular, in patients with periodontitis, supports the validity of our findings.

In conclusion, this study suggests that individuals with periodontitis carrying the minor alleles of *PADI2* SNPs rs2057094, rs2076616 or rs2235912 may be at increased risk of anti-CCP-negative RA irrespective of smoking status and at increased risk of anti-CCP-positive RA if they have a history of smoking. Thus, our data point towards a role for citrullination in the pathogenesis of not only anti-CCP-positive RA, but also anti-CCP-negative RA, particularly in individuals with periodontitis. This may have implications for PAD inhibitor therapy, which is currently under investigation as a novel treatment modality in anti-CCP-positive RA.

## 4. Materials and Methods

### 4.1. Patients and Controls

The study included stored blood samples from a cohort previously reported on by Pedersen et al. [31] and ourselves [30]. It contained 434 patients with RA diagnosed within the previous 5 years before recruitment from rheumatology and internal medicine departments throughout Denmark, which has a predominantly Caucasian population. The RA case definition was age between 18 and 65 years and fulfilment of the 1987 American College of Rheumatology (ACR) classification criteria for RA [40]. The control group was frequency-matched by sex and birth year, randomly selected from the Danish population by means of the Civil Registration System, a national database keeping track of all demographic changes in Denmark [31], and consisted of 524 subjects who did not fulfill the RA case definition. At study design, the case-control ratios were set at 1:1 for women and at 1:2 for men to enhance statistical power in analyses in the men subgroup. However, all invited subjects agreeing to participate were included. All participants underwent a computer-assisted 30 min telephone interview aimed to collect self-reported data on exposure and confounder variables, including smoking status. As for self-reported periodontitis, participants were asked if their dentist had ever told them that they had periodontitis and if so, how old they were when this was first observed. Notably, while self-reported periodontitis is a generally accepted measure in epidemiological studies [39], it may not be in complete accordance with the diagnosis obtained by clinical periodontal examination, and age at disease onset may be subject to recall bias. Detailed self-reported data on smoking habits were dichotomized as ever or never smoking. Anti-CCP status was determined prior to this study via a second-generation enzyme-linked immunosorbent assay (ELISA) using the Immunoscan RA kit (Euro-Diagnostica AB, Malmö, Sweden). The study was approved by the Scientific Ethical Committees for Copenhagen and Frederiksberg (KF 01-039/01) and the Danish Data Protection Agency (2001-41-0658), and written informed consent was obtained from all study subjects [31].

### 4.2. SNP Selection

**Figure 2:**
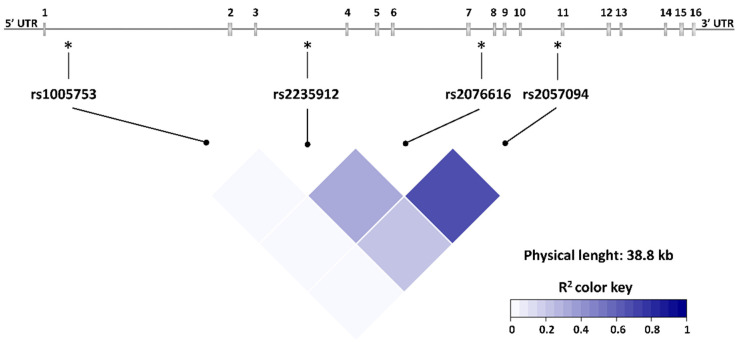
*PADI2* SNPs location in the gene and linkage disequilibrium heatmap. UTR, Untranslated region, 1–16 indicates the exon number. * indicates SNP location within the *PADI2* gene.

Based on previous reports on *PADI* SNPs in RA [26,27,28,29], the four intronic SNPs rs2057094, rs2076616, rs2235912 and rs1005753 in *PADI2* (Figure 2), and rs74058715 (5′ UTR), rs11203367 (exon missense), rs1748033 (exon synonymous) and rs2240335 (exon synonymous) in *PADI4* were included in our analyses. We chose rs11203367 as tag-SNP for a block of *PADI4* SNPs in high LD including rs11203366, rs11203367, rs874881, rs2240340, and rs11203368 (r^2^ > 0.8) [41]. The DNA was isolated from whole blood samples and stored at −80 °C until use as described [42].

### 4.3. Genotyping

Genotyping of samples was carried out with an in-house-developed multiplex SNP assay protocol, as previously described [43]. In brief, the selected SNP sites were amplified via polymerase chain reaction (PCR), and allele-specific primer extension (ASPE) oligonucleotides were labelled in an ASPE reaction prior to hybridization to MagPlex-TAG™ bead sets (Luminex Corporation, Austin, TX, USA) for analysis on the Luminex platform (Luminex Corporation, Austin, TX, USA). Control samples with known genotypes (Coriell Cell Repository, Camden, NJ, USA) as well as no-template negative controls were included in every run. Approximately 5% of the samples were randomly re-typed to confirm the obtained genotypes.

### 4.4. Statistical Analysis

All analyses were performed in RStudio Version 1.0.153 (RStudio Inc., Boston, MA, USA) software with R version 3.4.2 (R Foundation for Statistical Computing, Vienna, Austria). Possible deviations from Hardy-Weinberg equilibrium were calculated with the ‘hwde’-package (R Foundation for Statistical Computing, Vienna, Austria). The LD between SNPs in either *PADI2* or *PADI4* was calculated with the “LDheatmap” package in R, using SNPs locations according to the Genome Reference Consortium GRCh38.p13 genome assembly [44]. Trend tests for minor allele counts to assess associations between *PADI* SNPs and risk of RA were performed by multiple logistic regression analyses with adjustment for potential confounders such as age, sex, and smoking status. *p*-values < 0.05 were accepted as statistically significant.

## Figures and Tables

**Table 1:** Demographic and clinical characteristics of anti-citrullinated protein antibody (anti-CCP)-positive and anti-CCP-negative rheumatoid arthritis (RA) and non-RA controls.

		Anti-CCP-Positive RA (*n* = 300)	Anti-CCP-Negative RA (*n* = 134)	Controls (*n* = 524)
Age at study inclusion, median (range), years		52 (19–68)	53 (21–69)	53 (19–69)
Women, *n* (%)		202 (67%)	103 (77%)	321 (61%)
Ever smoker, *n* (%)		222 (74%)	83 (62%)	319 (61%)
European ancestry (%)		99%	99%	99%
Self-reported periodontitis, *n* (%)		93 (31%)	44 (33%)	161 (31%)
Age at periodontitis onset, median (range), years		38 (7–61) *	35 (10–65) **	35 (8–66) ***
Age at RA diagnosis, median (range), years		51 (18–65)	50 (22–66)	NA
HLA-DRB1*04	Self-reported periodontitis yes, *n* (%)	68 (73.1%)	12 (27.3%)	52 (32.3%)
	Self-reported periodontitis no, *n* (%)	153 (73.9%)	36 (40%)	136 (37.5%)
HLA-DRB1*01	Self-reported periodontitis yes, *n* (%)	15 (16.1%)	5 (11.4%)	29 (18%)
	Self-reported periodontitis no, *n* (%)	63 (30.4%)	22 (24.4%)	71 (19.6%)

* n = 85 ** *n* = 39 *** *n* = 146. NA: Not applicable.

**Table 2:** Associations between polymorphisms in the peptidylarginine deiminase 2 gene (*PADI2*) and rheumatoid arthritis (RA), in general (*n* = 137), anti-CCP-positive RA (*n* = 93) or anti-CCP-negative RA (*n* = 44) in particular, in subjects with self-reported periodontitis (*n* = 298).

	pp	pq	qq	OR (Trend Test)	95% CI	*p*-Value
**rs2057094**	**TT**	**TC**	**CC**			
**Population controls**	66	69	26			
**RA**	41	67	29	**1.42**	**[1.02; 1.98]**	**0.03**
**Anti-CCP-positive RA**	31	44	18	1.28	[0.89; 1.84]	0.18
**Anti-CCP-negative RA**	10	23	11	**1.75**	**[1.08; 2.84]**	**0.02**
**rs2076616**	**TT**	**TC**	**CC**			
**Population controls**	84	58	19			
**RA**	60	56	21	1.35	[0.96; 1.88]	0.08
**Anti-CCP-positive RA**	44	37	12	1.21	[0.83; 1.76]	0.32
**Anti-CCP-negative RA**	16	19	9	**1.67**	**[1.04; 2.68]**	**0.04**
**rs2235912**	**GG**	**GC**	**CC**			
**Population controls**	67	69	25			
**RA**	39	70	28	**1.48**	**[1.06; 2.07]**	**0.02**
**Anti-CCP-positive RA**	29	49	15	1.28	[0.88; 1.86]	0.20
**Anti-CCP-negative RA**	10	21	13	**2.04**	**[1.25; 3.34]**	**0.004**
**rs1005753**	**TT**	**TG**	**GG**			
**Population controls**	61	80	20			
**RA**	43	76	18	1.12	[0.79; 1.60]	0.52
**Anti-CCP-positive RA**	28	54	11	1.14	[0.76; 1.70]	0.52
**Anti-CCP-negative RA**	15	22	7	1.06	[0.64; 1.77]	0.82

p: Major allele, q: Minor allele, OR: Odds ratio, CI: Confidence interval. Logistic regression with adjustment for age, sex, and smoking status. Values in bold indicate *p* < 0.05.

## Data Availability

The datasets generated during and/or analyzed during the current study are available from the corresponding author on reasonable request.
